# Distinct Tomato Cultivars Are Characterized by a Differential Pattern of Biochemical Responses to Drought Stress

**DOI:** 10.3390/ijms23105412

**Published:** 2022-05-12

**Authors:** Veronica Conti, Claudio Cantini, Marco Romi, Maria Michela Cesare, Luigi Parrotta, Stefano Del Duca, Giampiero Cai

**Affiliations:** 1 Department of Life Sciences, University of Siena, 53100 Siena, Italy; marco.romi@unisi.it (M.R.); maria.cesare@student.unisi.it (M.M.C.); giampiero.cai@unisi.it (G.C.); 2 National Research Council of Italy, Institute for Bioeconomy (CNR-IBE), 58022 Follonica, Italy; claudio.cantini@ibe.cnr.it; 3 Department of Biological, Geological and Environmental Sciences, University of Bologna, 40126 Bologna, Italy; luigi.parrotta@unibo.it (L.P.); stefano.delduca@unibo.it (S.D.D.); 4 Interdepartmental Centre for Agri-Food Industrial Research, University of Bologna, 47521 Cesena, Italy

**Keywords:** HSP70, CYP, osmotin, dehydrin, aquaporins, RuBisCO, SuSy

## Abstract

Future climate scenarios suggest that crop plants will experience environmental changes capable of affecting their productivity. Among the most harmful environmental stresses is drought, defined as a total or partial lack of water availability. It is essential to study and understand both the damage caused by drought on crop plants and the mechanisms implemented to tolerate the stress. In this study, we focused on four cultivars of tomato, an economically important crop in the Mediterranean basin. We investigated the biochemical mechanisms of plant defense against drought by focusing on proteins specifically involved in this stress, such as osmotin, dehydrin, and aquaporin, and on proteins involved in the general stress response, such as HSP70 and cyclophilins. Since sugars are also known to act as osmoprotectants in plant cells, proteins involved in sugar metabolism (such as RuBisCO and sucrose synthase) were also analyzed. The results show crucial differences in biochemical behavior among the selected cultivars and highlight that the most tolerant tomato cultivars adopt quite specific biochemical strategies such as different accumulations of aquaporins and osmotins. The data set also suggests that RuBisCO isoforms and aquaporins can be used as markers of tolerance/susceptibility to drought stress and be used to select tomato cultivars within breeding programs.

## 1. Introduction

Global warming, primarily due to the intense emission of carbon dioxide caused by human activities, is constantly increasing the average temperature; this is responsible for the reduction of rainfalls in highly vulnerable areas such as the Mediterranean, which is considered a “hot spot” in the 21st century [1]. It is unlikely that the world will experience an inversion in climate changes, especially in the coming years. It therefore becomes pressing to determine the effects that water scarcity can cause at the level of natural and anthropogenic ecosystems. Considering that modern agriculture uses copious quantities of water [2], the impact of water scarcity on the productivity of cultivated plants and the relative costs must not be underestimated.

At the level of individual plant organisms, water scarcity is perceived as an abiotic stress (drought stress). Drought stress leads to various damages in plants, including incorrect protein folding, alterations in enzymatic functions and higher production of reactive oxygen species (ROS) [3]. The first response of plants to water deficit consists of stomata closure mediated by abscisic acid (ABA) [4]. ABA has a key role in the control of ABA-dependent gene transcription, allowing the production of proteins specifically targeted to counteract drought stress [5]. Parallel to the production of specific hormones, other physiological/biochemical defensive activities contribute to make plants more tolerant to water stress. For instances, production of osmoprotectants (which prevent proteins from denaturing and retain water in the cells) [6] and the enhanced activity of antioxidants (that reduce the levels of ROS [7]) are relevant.

In general, abiotic stresses are associated with enzyme and protein dysfunction and it is important for the plant to keep the cells functional during stress. For this purpose, plants increase the expression of various genes under drought stress leading to the production of corresponding proteins. Most importantly, it is necessary to prevent the aggregation of non-native proteins and to maintain the proteins in their functional conformation. Therefore, some of the most important proteins induced by drought stress include chaperones, such as heat shock proteins (HSPs), and abundant proteins of late embryogenesis (LEA) [8].

Among the proteins induced by ABA are Heat Shock Proteins (HSPs), a class of chaperones involved in protein folding and thus relevant in the defense against abiotic stresses [9,10]. HSPs are expressed especially in heat stress conditions, but also in case of drought, salinity stress and pathogen infections [11,12,13]. HSPs of 70 kDa (HSP70) are more involved in tolerance to heat and drought stresses [14].

Cyclophilins (CYPs) are another ubiquitous chaperone proteins, involved in a wide range of cellular processes [15,16,17]. CYPs are peptidyl-prolyl cis-trans isomerases and catalyze the cis-trans isomerization of the amide bond between a proline residue and the previous amino acid residue, a step critical for the correct protein folding [18]. Due to their catalytic activity, CYPs can accelerate the folding of different proteins in response to various biotic and abiotic stresses [19,20].

The synthesis of Late Embryogenesis Abundant (LEA) proteins is also induced by ABA [21]. Dehydrins belong to the family II of LEA proteins and are involved in the plant’s response to dehydration and, more generally, to abiotic stresses [22]. Dehydrins can protect the activity of proteins by preventing their denaturation [23,24]. In addition, they can bind to membrane phospholipids, such as phosphatidic acid, whose level increases in response to ABA [25]. Moreover, in the presence of zinc ions, dehydrins bind to DNA, which is therefore repaired or protected from damage caused by environmental stresses, as observed in Japanese mandarins [26].

Abiotic stresses activate many intracellular signals which lead to the accumulation of osmoprotectants and production of Pathogenesis-related (PR) proteins [27]. Osmotin is a 24 kDa protein belonging to the PR-5 family. In addition to significant antifungal and antibacterial activity, it can increase plant resistance to various abiotic stresses, such as salt and drought stress [28]. Osmotin also induces the expression of genes involved in proline biosynthesis, causing its accumulation within cells and providing plants with increased tolerance to drought stress [29,30,31,32]. Moreover, osmotin can protect chlorophyll and the photosynthetic machinery in conditions of water scarcity [33].

Proteins important for the efficiency of photosynthesis are also the aquaporins. These are known as water and CO_2_ transporters [34]. According to their structure and localization, aquaporins are classified into five groups; the plasma membrane intrinsic proteins (PIPs) are the ones most involved in CO_2_ and H_2_O transport. Indeed, some work has shown that overexpression of PIPs in Arabidopsis, rice, or tobacco results in enhanced CO_2_ assimilation in leaves [35,36,37]. Furthermore, overexpression of PIPs in several cultivated plants led to a better response to drought stress [38]. For example, overexpression of a PIP1;2 in bananas increased tolerance to both drought and salt stress [39]. In tomatoes, overexpression of PIPs also resulted in improved drought stress tolerance [40,41].

Other biochemical adaptations of plants consist of the regulation of the photosynthetic process. Photosynthesis might be limited due to the scarce availability of substrates such as H_2_O and CO_2_. In the dark phase (the Calvin cycle), the leading enzyme is Ribulose 1,5-Bisphosphate Carboxylase/Oxygenase (RuBisCO) which catalyzes the carboxylation reaction that initiates CO_2_ fixation. The catalytic activity of RuBisCo progressively decreases with increasing duration and severity of drought conditions [42]. This can be explained by a partial loss of the protein during stress [43]. The degradation of RuBisCO generates fragments of the enzyme, which can be detected by two-dimensional electrophoresis [44]. Furthermore, RuBisCO, through the carboxylation reaction, generates substrates for the synthesis of sucrose, which is crucial for the growth of plants. Therefore, any damage to RuBisCo activity or quantity has significant impacts on plant biomass.

At the level of sink tissues, sucrose can enter cells in two ways. It can be split by the activity of cell wall invertases into glucose and fructose, which are transported into cells by monosaccharide transporters [45]. Sucrose can also enter cells directly through the activity of sucrose transporters. Once in the cells, sucrose can be cleaved by both soluble invertase but also by sucrose synthase (SuSy) with energetically different results [46,47]. SuSy activity is relevant under drought stress conditions because it saves energy in the UDP-glucose molecule and because it increases the concentration of hexose sugars. The latter are precious osmoprotectants and detoxifying molecules with a key role in plant’s protection against oxidative stress [48].

In this manuscript, we analyzed the biochemical response of tomato cultivars subjected to drought stress. Tomato is a widely cultivated plant that can suffer dramatically from water stress conditions [49]. Although the genetic response of tomato to drought stress is partly known [50,51,52], the involvement of specific proteins in defense to water shortage still needs to be carefully evaluated. It should also be considered that different tomato cultivars exhibit different biochemical responses in relation to their specific genetic background. In previous works we have already observed that locally adapted Tuscan tomato cultivars exhibit different responses in physiological terms [53,54], as well as in the content of polyphenols and antioxidants at the fruit level [55]. 

The purpose of this work was to test the hypothesis that drought susceptibility or tolerance of tomato cultivars are based on the different production of metabolic proteins, such as RuBisCO and SuSy that regulate the level of osmoprotective sugars, and on the change in the content of proteins specifically involved in the stress response, such as HSP70, cyclophilins, osmotin, dehydrin, and aquaporins.

## 2. Results and Discussion

In this manuscript, we focused on four tomato cultivars characteristic of the Tuscany Region (Italy). This manuscript is based on previous works [54] in which a larger number of cultivars had been selected and analyzed for drought stress tolerance. The data collected previously allowed us to define the tolerance/susceptibility profile of tomato cultivars, thereby identifying a few cultivars of major interest, i.e., the four studied in this manuscript: the most resistant (Perina and Fragola), the most susceptible (Pisanello), and the one with intermediate resistance traits (Quarantino). The choice to focus the analysis on four tomato cultivars (compared to the previous 13 case studies) was driven by the attempt to investigate cultivars with distinct characteristics. The protein analysis, as well as the previously performed studies, helps to define the tolerance/susceptibility profile of the four selected cultivars. We chose to analyze proteins involved in general stress responses (such as HSP70 and cyclophilin), in drought resistance (such as dehydrins, osmotin, aquaporins), and in more strictly metabolic aspects (RuBisCO and sucrose synthase).

### 2.1. Levels of HSP70 Increase after Drought Stress

**Figure 1:**
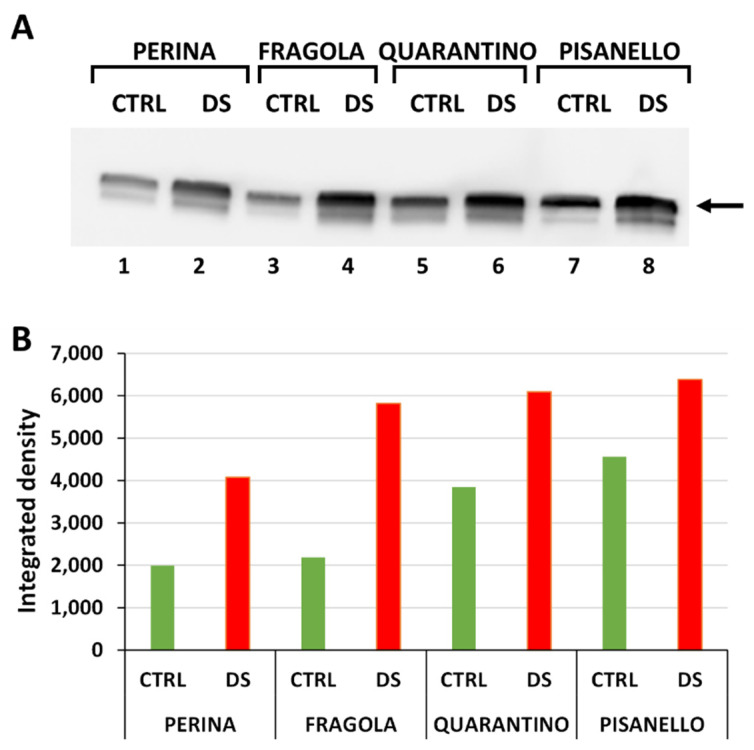
Content of HSP70 in leaves of the four tomato cultivars from both control (CTRL) and stressed (DS) samples. (**A**) Immunoblotting with anti-HSP70 antibody. The arrow indicates the band with molecular weight between 70–75 kDa. Lane 1, Perina CTRL; lane 2, Perina DS; lane 3 Fragola CTRL; lane 4, Fragola DS; lane 5, Quarantino CTRL; lane 6 Quarantino DS; lane 7, Pisanello CTRL; lane 8, Pisanello DS. Here and in all subsequent gels an equal amount of protein (40 µg) was loaded into all lanes. (**B**) Quantitation of the relative content of individual bands in different samples. Green bars indicate control samples, red bars those that are drought stressed.

We initially examined the general response to drought stress by evaluating the relative content of HSP70 (Figure 1A). Expression of the protein is detectable in each of the 8 samples examined. This result shows constitutive basal-level expression of HSP70 even in samples from plants that were irrigated normally. Figure 1B is the densitometric analysis carried out on the HSP70 bands detected after immunoblotting. Levels of HSP70 increase in stressed plants compared with controls for each cultivar. This finding is not surprising because many studies have reported that under abiotic stress conditions the content of HSP70 increases to protect protein structure and cell membranes as well as to counteract the increase in ROS levels [10,13]. In addition, a direct correlation has been observed between drought stress and accumulation of HSP70 [14]. 

Comparing the content of HSP70 in DS samples of the four cultivars, the increase in protein levels from the most tolerant to the most susceptible cultivar is evident. The cultivar Perina showed lower content than the others, especially compared to Pisanello. Even in the CTRL samples, the content of HSP70 increased from the most tolerant to the most susceptible cultivar. Furthermore, in the control samples, basal levels of HSP70 in Perina are lower than in Quarantino or Pisanello. Therefore, results cannot be interpreted only in terms of quantity, but it is necessary to compare the increase of HSP70 from CTRL to DS. Both the cultivars Perina and Fragola increase the content of HSP70 more than 200% in DS plants compared to those in the CTRL group, whereas in Quarantino, HSP70 levels increase about 50%. In contrast, Pisanello, while showing an abundant basal content of HSP70, only reveals a smaller increase than the other cultivars. Therefore, it is inferred that the increase in content of HSP70 in Pisanello under drought stress is the lowest among all cultivars.

Consistent with morpho-physiological analysis [54], the Fragola and Perina cultivars experience less lack of water as stress due to a more efficient molecular response. The opposite occurs for Pisanello, which shows unstable and ineffective responses to drought stress from the first days of treatment. These results confirm the protective role of HSP70 in drought and osmotic stress and show that the most tolerant tomato cultivars respond by increasing the HSP70 content to restore cell function and recover from drought stress. An increase in HSP70 content has been repeatedly observed under abiotic stress conditions, for example in tomatoes subjected to high temperatures [56] and in sugarcane under drought stress conditions [14]. Although the increase in HSP70 under abiotic stress is not actually surprising, the difference observed between tomato cultivars is noteworthy; this again highlights how different genotypes can exhibit different responses in terms of chaperone proteins.

### 2.2. Cyclophilin Levels Also Trend Upward in Drought-Stressed Cultivars

**Figure 2:**
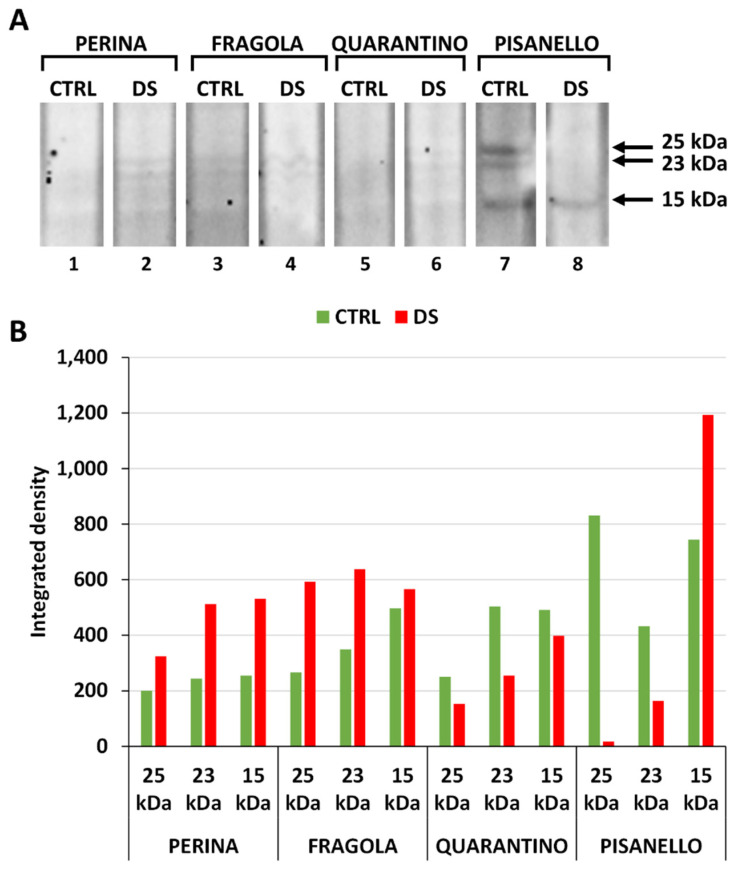
Content of the cyclophilin family in the four tomato cultivars in both control (CTRL) and drought-stressed (DS) samples. (**A**) Immunoblot analysis of cyclophilin with the three bands identified at 25, 23, and 15 kDa. Lane 1, Perina CTRL; lane 2, Perina DS; lane 3 Fragola CTRL; lane 4, Fragola DS; lane 5, Quarantino CTRL; lane 6 Quarantino DS; lane 7, Pisanello CTRL; lane 8, Pisanello DS. (**B**) Quantitative analysis of the relative content of the three cyclophilin bands in the different samples. In green the control samples, in red the stressed samples.

As a further general analysis of the stress response, we subsequently investigated cyclophylins (CYP). Figure 2A shows that the anti-cyclophilin antibody cross-reacts with at least three polypeptides (at 25, 23 and 15 kDa) in the stressed and control plant samples of each cultivar. Blottings were also subjected to densitometric analysis (Figure 2B) showing that cyclophilin levels increased in drought-stressed samples compared to controls. The levels of all three cyclophilin bands in the DS group of the cultivar Perina increased relative to CTRL. The same applies to the Fragola cultivar, although in a less pronounced way. In contrast, the intensity of bands in the DS sample of Quarantino cultivar decreases relative to CTRL. Finally, the cultivar Pisanello behaves in a peculiar way, by drastically decreasing the amount of the 25 kDa and 23 kDa bands but increasing the amount of the 15 kDa band.

The cultivars Perina and Fragola were selected in previous studies based on their tolerance to drought stress; however, the cultivar Quarantino showed both traits of tolerance and susceptibility [53,54]. The higher content of cyclophilins in the drought-stressed cultivars Perina and Fragola compared to Quarantino is consistent with the study by Barik and coworkers [20]. A correlation between cyclophilins and resistance to abiotic stresses has been described in several cases; for example, in Arabidopsis, cyclophilin encoded by the ROC3 gene is positively correlated with resistance to drought stress, as cyclophilin Roc3 likely regulates the levels of reactive oxygen species and stomatal opening [57]. A correlation between drought stress and increased expression of specific cyclophilins was also found in wheat [58]. The role of cyclophilins in drought tolerance, as well as other abiotic stresses, is further supported by the overexpression of a pigeonpea gene in transgenic Arabidopsis plants, which consequently acquired more tolerance to abiotic stresses [59]. Also in sorghum, application of drought stress induced expression of a 20-kDa cyclophilin in a cultivar-dependent manner [60]. 

In rice, drought and salt stress induce considerable expression of a specific cyclophilin; furthermore, overexpression of this protein in transgenic rice and Arabidopsis plants increased drought tolerance [61]. Therefore, we hypothesize that the tolerance of tomato cultivars is due to a high expression of cyclophilins, which accelerate the process of protein folding under stress conditions. The low stress tolerance of the Pisanello cultivar is likely due to down-regulation of transcription of the 25- and 23-kDa cyclophilin genes, which could encode for important protective factors.

### 2.3. Cultivars under Drought Stress Exhibit a Significant Increase in Dehydrin Levels

**Figure 3:**
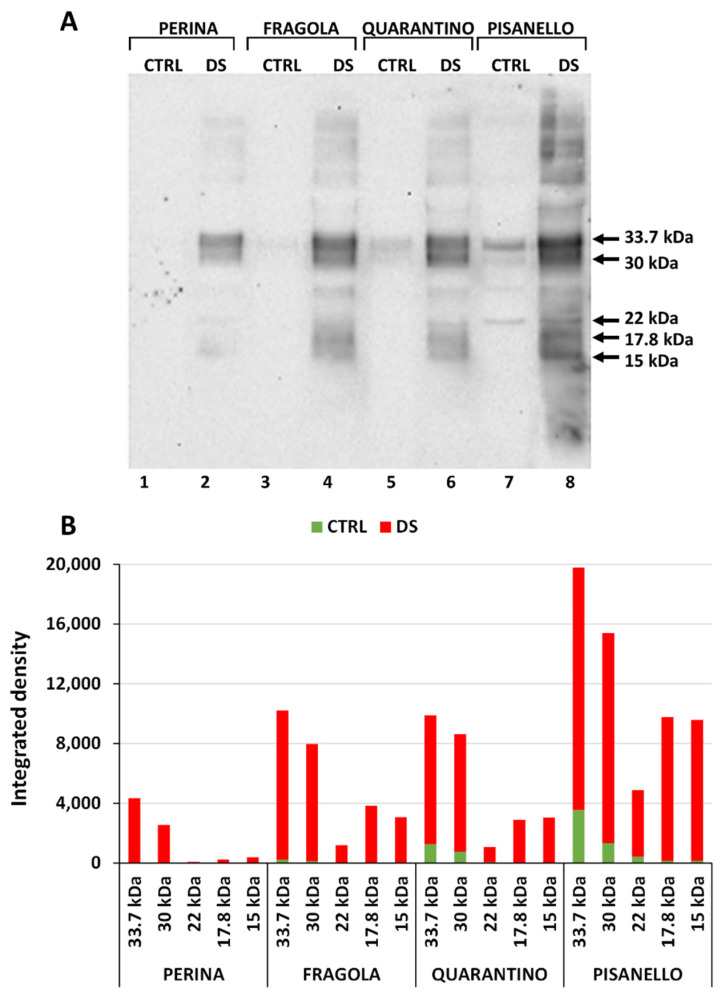
Content of dehydrins in both control and stressed plants of the four tomato cultivars. (**A**) Immunoblotting in leaves of the four tomato varieties analyzed. Lane 1, Perina CTRL; lane 2, Perina DS; lane 3 Fragola CTRL; lane 4, Fragola DS; lane 5, Quarantino CTRL; lane 6 Quarantino DS; lane 7, Pisanello CTRL; lane 8, Pisanello DS. The major dehydrins identified have molecular weights of 33.7, 30, 22, 17.8, and 15 kDa. (**B**) Relative content of dehydrins in the four tomato cultivars in both control (CTRL, in green) and drought-stressed (DS, in red) samples. Please note that in this graph the green bars of control samples are superimposed on the red bars of stressed samples.

After depicting a preliminary picture of the overall response to drought stress, we moved on to the analysis of proteins more specifically involved in the stress response. Figure 3A shows the blotting performed with the anti-dehydrin antibody; five polypeptide bands with molecular weight of 33.7, 30, 22, 17.8, and 15 kDa can be highlighted. The presence of numerous bands was predictable because dehydrins are a family of proteins classified into at least five different structural groups based on the number and order of three conserved distinctive motifs, the K, Y, and S segments [62]. The blot shows that dehydrins’ content increases significantly under drought stress conditions. This result is expected and consistent with findings by Borovskii and coworkers [63] who demonstrated the relationship between dehydration and increased dehydrin levels.

Furthermore, the increase in dehydrin content in stressed samples is inversely proportional to the tolerance of cultivars toward drought stress (Figure 3B). The intensity of bands increases significantly from the cultivar Perina (i.e., the most tolerant) to the cultivar Pisanello, namely the most susceptible to stress [54]. This result can be compared with that obtained by Velasco-Conde and coworkers [64], in which a drought-resistant variety of pine (Pinus pinaster) expressed higher amounts of dehydrins when subjected to drought stress. Dehydrins are proteins traditionally associated with resistance against drought and other stressful conditions. In fact, they can increase water retention capacity, have positive effects on chlorophyll content, preserve the photosynthetic machinery and increase detoxification of reactive oxygen species while promoting the accumulation of compatible solutes [65]. Supporting data were also obtained in soybean; dehydrins of 28 and 32 kDa were found after water deprivation in developing seeds, but not in seeds from well-watered plants [66]. A case comparable to our findings was described in wheat, where analysis of several cultivars revealed accumulation of a specific 24-kDa dehydrin in distinct cultivars under water stress, whereas no accumulation was detected in control wheat plants [67]. Different soybean varieties under drought stress also showed distinct accumulation of specific antibody-detected dehydrins; this again emphasizes that varietal response can be quite distinctive [68]. In the case of tomato cultivars, Pisanello (the most susceptible) likely require higher amounts of dehydrins to cope with stress damage. On the contrary, the most tolerant genotypes do not suffer particularly severe damages and produce less dehydrins than the most susceptible cultivars. Most notably, an increase in the 33.7 and 30 kDa bands is found in the case of Perina and Fragola; supposedly those are the dehydrins most used to control drought stress.

### 2.4. Osmotin Levels Increased Only in the Pisanello Cultivar under Drought Stress

**Figure 4:**
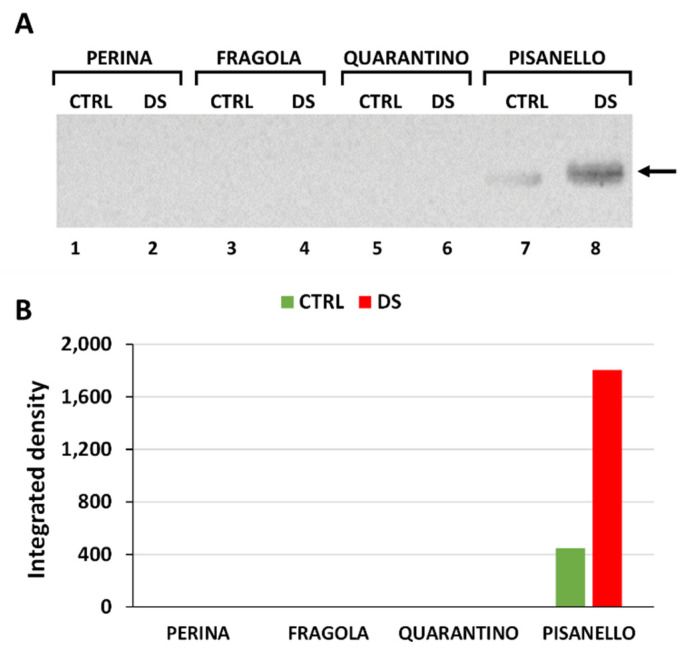
Content of osmotin in the leaves of the four tomato cultivars. (**A**) Immunoblotting with the anti-osmotin antibody. Lane 1, Perina CTRL; lane 2, Perina DS; lane 3 Fragola CTRL; lane 4, Fragola DS; lane 5, Quarantino CTRL; lane 6 Quarantino DS; lane 7, Pisanello CTRL; lane 8, Pisanello DS. The arrow indicates the position of the only immunoreactive band. (**B**) Relative quantization of the immunoblotting signal in controls (CTRL, green bar) and stressed samples (DS, red bar).

To cope with abiotic stresses, plants have several defense mechanisms including the protein osmotin, which belongs to the PR-5 family of pathogenesis-related (PR) proteins [69]. The blot in Figure 4A shows detectable levels of osmotin only for the cultivar Pisanello in both the CTRL and DS samples. The other three cultivars, namely Perina, Fragola, and Quarantino, which exhibited higher drought tolerance, did not show immunoreactive bands. It is likely that the Pisanello cultivar, being the most susceptible to drought and the most damaged in terms of photosynthesis, is the only cultivar that needs the expression of osmotin. Indeed, this protein has a protective activity against chlorophyll and the entire photosynthetic apparatus in case of osmotic stress [33]. Densitometric analysis in Figure 4B showed that the osmotin levels of the Pisanello cultivar are significantly higher in DS samples than in CTRL samples. This data is consistent with and confirms previous studies in tomatoes [70,71] where osmotin production was found to be induced by endogenous levels of ABA and therefore by severe drought. The importance of osmotin is also demonstrated by transgenesis experiments in which the tobacco osmotin gene was expressed in tomato plants. The results showed increased tolerance to salt and drought stresses in transgenic plants, with higher relative water content, higher chlorophyll, and proline content [28]. The same protective effect exerted by overexpressing the tobacco osmotin gene in tomato plants was also observed in response to cold treatment, suggesting that osmotin is important in all conditions related to less water uptake [72]. The results show that osmotin is a highly discriminating protein for selected tomato cultivars, especially regarding Pisanello, the most susceptible one. It is not clear why only the Pisanello cultivar should express osmotin in a stress-dependent manner. The only reasonable conclusion is that the other more tolerant cultivars do not need to implement this protective mechanism. Only the cultivar Pisanello, which is defective in other responses to drought stress, therefore increased the expression of osmotin to counteract the deleterious effects of stress.

### 2.5. Aquaporins

**Figure 5:**
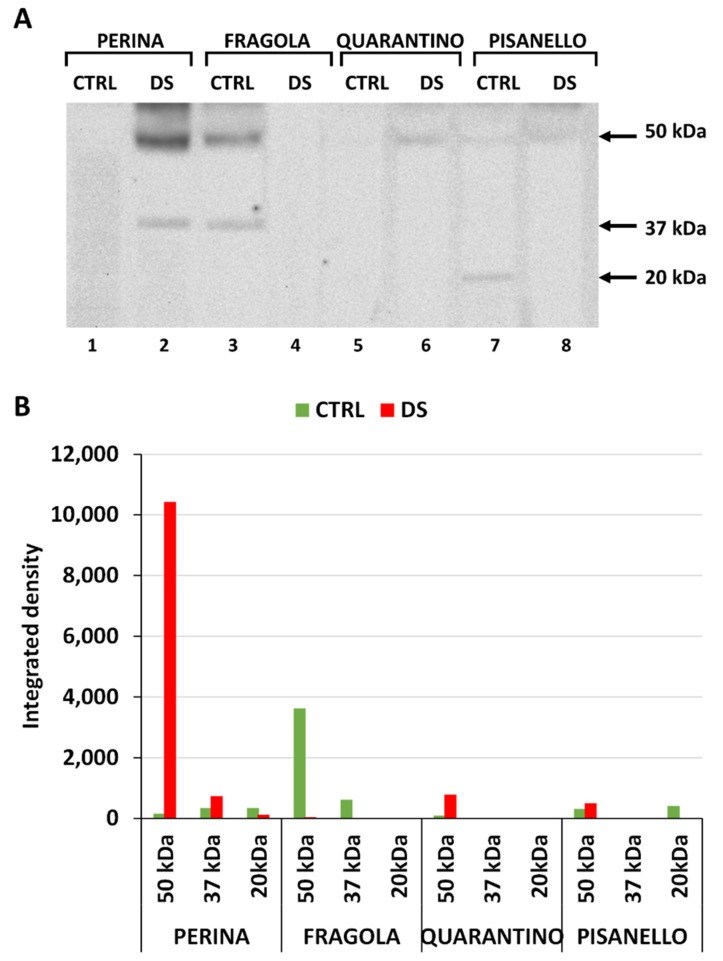
Aquaporin content in leaves of the four tomato cultivars under both control and stressed conditions. (**A**) Immunoblotting with anti-aquaporin antibodies in the four cultivars, control samples (CTRL), and stressed samples (DS). On the right, molecular weights of the three main bands identified. (**B**) Relative quantization of blotting expressed as integrated density (y-axis). Green bars indicate control samples, red bars indicate drought-stressed samples.

Aquaporins are proteins located in the plasma and intracellular membrane and are well known transporters of H_2_O and CO_2_, two important substrates for photosynthesis [34]. The blot in Figure 5A shows quite different protein content between cultivars. Three major immunoreactive bands were identified, at 50, 37, and 25 kDa. Bands were not present in all samples but showed an extremely specific distribution with respect to both individual cultivars and molecular weights. Densitometric analysis in Figure 5B shows a clear increase of the 50-kDa aquaporins in the Perina DS cultivar compared to its control. On the contrary, the cultivar Fragola is characterized by a decrease of both 50- and 37-kDa aquaporins in stressed samples. Finally, in Quarantino and Pisanello no relevant difference between the CTRL and DS samples is evident. 

The role of aquaporins in plants under drought conditions has not yet been fully investigated. Aquaporins have been related to salt stress in tomatoes [73], as well as in relation to plant-fungus interactions in mycorrhizae [74]. The expression of aquaporins in tomato seeds was also related to the specific irradiation light and the presence of metals such as mercury [75]. However, given their role as water transporters, aquaporins are likely involved in several physiological processes, such as the movement of water and solutes that results in the control of stomata opening and maintenance of hydraulic conductance between roots, stems, and leaves [76]. Consequently, a correlation between the expression of aquaporins and plant susceptibility or tolerance to drought stress is expected [77]. This is also confirmed by genetic analyses showing that the expression of aquaporins is related to a higher tolerance to drought stress in tomatoes [78].

Forty-seven genes encoding for aquaporins have been identified in tomatoes. As for the family of plasma membrane intrinsic proteins (PIPs), among the five forms recognized by the antibody we used only one is present in mature tomato leaves, namely PIP1;3. The PIP1;1 protein is strongly expressed in roots and fruits, PIP1;2 in the young leaf and root, PIP1;5 only during fruit development, and PIP1;4 is not present in tomatoes [79]. In addition to the role as water carriers, PIP family members facilitate the diffusion of CO_2_ in the mesophyll [35,37]. Considering the data and results obtained, it can be concluded that the higher expression of PIPs in the Perina cultivar might allow for higher stress tolerance; this is likely related to increase of both CO_2_ and H_2_O transport and more efficient photosynthesis. This hypothesis is also strengthened by work on transgenic tomato plants expressing a drought-inducible aquaporin gene PIP1;3, which derived from Malus domestica Borkh [41]. The tomato plants exhibited a slower rate of water loss than the wild type and stomata closed faster in response to drought.

### 2.6. RuBisCO Levels Decrease Significantly in the Pisanello Cultivar While the Four Cultivars Make Differential Use of RuBisCO Isoforms

Ribulose 1,5-bisphosphate carboxylase/oxygenase (RuBisCO) is the first enzyme in the Calvin-Benson cycle. It catalyzes the carboxylation reaction that initiates CO_2_ fixation. This enzyme can account for up to 50% of the protein content in a leaf. There is no need to emphasize that it is an extremely critical enzyme in the metabolism and life of plants and that any alteration in the levels or activity of this enzyme has major consequences in the production of plant biomass. 

**Figure 6:**
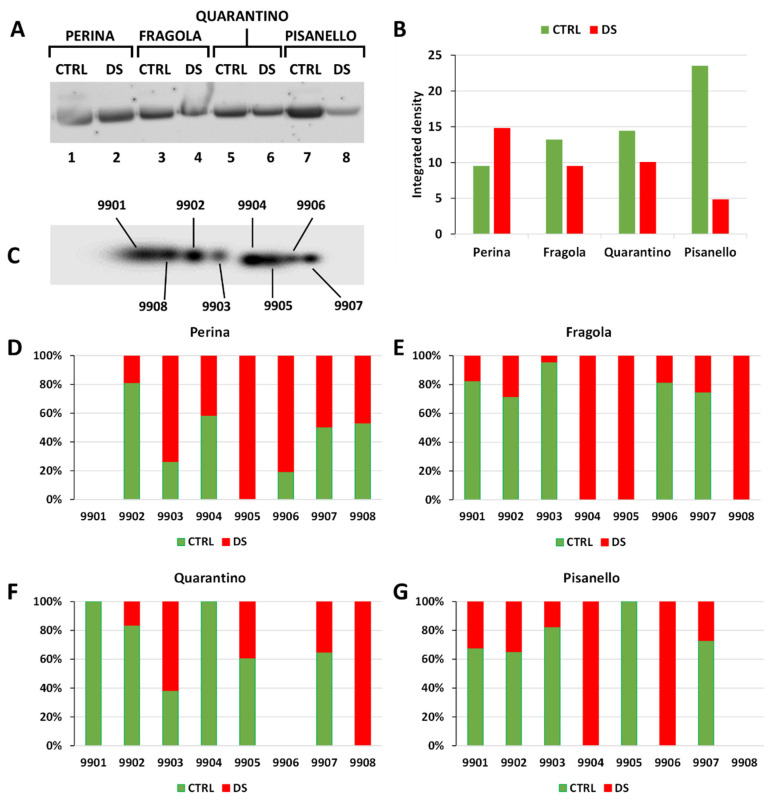
Content and isoform composition of RuBisCO in control and stressed plants of the four tomato cultivars. (**A**) 1D immunoblotting of RuBisCO in the four cultivars analyzed. Lane 1, Perina CTRL; lane 2, Perina DS; lane 3, Fragola CTRL; lane 4, Fragola DS; lane 5, Quarantino CTRL; lane 6 Quarantino DS; lane 7, Pisanello CTRL; lane 8, Pisanello DS. (**B**) Relative quantitative analysis of 1D immunoblotting in both control (green bar) and stressed samples (red bar). (**C**) Master (virtual) blot of RuBisCO isoforms after 2D electrophoresis. Each sample contained 300 μg of protein. The blot contains all the spots detected by the anti-RuBisCO antibody, which are numbered automatically by the PDQuest software. Relative percentage content of RuBisCO isoforms in both control and stressed samples of Perina (**D**), Fragola (**E**), Quarantino (**F**) and Pisanello (**G**) cultivars. Again, green bars represent control samples and red bars indicate stressed samples.

Figure 6A shows the RuBisCO content in the leaves of the four cultivars as revealed by the antibody reaction. RuBisCO levels increase or only slightly decrease in the most tolerant cultivars. On the contrary, they decrease drastically in the most sensitive cultivar, Pisanello. Figure 6B confirms the visual data and highlights the drastic drop (about 80%) of RuBisCO in the Pisanello cultivar. As a term of comparison, the study by Hasanagić and coworkers [80] showed that RuBisCO decreases in tomato leaves after an extended period of drought stress. At the same time, the decrease in intracellular CO_2_ concentration caused by stomata closure induces an increase in the oxygenase activity of RuBisCO [81]. To initiate the process of photorespiration, the enzyme uses O_2_, which helps to keep the dark phase of photosynthesis active and protects chloroplasts from excessive ROS production. Under drought stress conditions, decreased transcription of genes encoding for small subunits of RuBisCO may occur, thus leading to the loss of enzyme stability [42]. In addition, the catalytic activity of RuBisCo progressively decreases with increasing duration and severity of drought conditions [42]; this may also be due to a partial loss of the protein because of stress.

Analysis by 2D electrophoresis and immunoblotting on RuBisCO revealed a series of spots, whose total number is shown in the virtual master blot of Figure 6C. The master blot is the sum of all spots present in the four cultivars under both control and drought stress conditions and provides a complete picture of all isoforms in the leaves of the tomato cultivars. The graphs below (Figure 6D–G) compare quantitatively the isoforms of RuBisCO in the four cultivars. The analysis revealed eight protein isoforms, but they differed among tomato cultivars. Loss of RuBisCO isoforms is associated with plant susceptibility to drought stress as reported, for example, in wheat [44] and sunflower varieties [82]. This may be explained by the degradation of the most susceptible isoforms or by the fact that plants modulate biosynthetic activity using the isoforms most adapted to drought conditions. Either of these explanations would imply that RuBisCO isoforms in DS groups correspond to those more resistant to water stress conditions. 

The 8 spots identified are more or less present in each case analyzed, at the level of cultivars as well as in the comparison between control and stressed samples. However, some substantial differences emerge. From a qualitative point of view, the differences between cultivars are minimal. Some cultivars are characterized by spots present only in stressed samples. For example, the cultivar Perina expresses the isoform 9905 only in the stressed sample. Even the cultivar Fragola shows a typical expression with the isoforms 9904 and 9905 being present only in the stressed samples. Others, such as the cultivar Quarantino, lack a specific spot, in this case 9906. Pisanello differs because some spots, such as 9904, are present only in the stressed sample, while 9905 is detected only in the control sample. Apart from the qualitative aspect, it is also noteworthy that the four cultivars differ in terms of the quality (usage) of individual spots. In the cultivar Perina, some isoforms are present correspondingly between control and stressed samples while others are typical of control samples (such as spot 9902) while 9905 is typical of stressed samples. The cultivar Fragola differs substantially from Perina because spots can be classified as almost exclusive to the control sample (spot 9901, 9902, 9906, 9907) and as exclusive to the stressed sample (spot 9904, 9905 and 9908). The cultivar Quarantino has a similar behavior to the Perina cultivar; the Pisanello cultivar is similar to the case of the Fragola cultivar with spots almost exclusive to the control sample and two spots (9904 and 9906) exclusive to the stressed sample. In the case of Pisanello, it is worth noting the absence of spot 9908.

In summary, the data indicate that the 9905 RuBisCO isoform is typical of the most tolerant cultivars (Perina and Fragola) and is therefore preferentially used; this isoform is partially expressed in Quarantino and is completely absent in the most susceptible cultivar (Pisanello). Since the large subunit of RuBisCO is encoded by a single plastidial gene, the different isoforms may be the result of post-translational modifications. Indeed, RuBisCO is characterized by several potential co-/post-translational modification sites [83]. It is assumed that upon stress modifications can generate RubisCO isoforms that are better suited to cope with a demanding situation. In support of this hypothesis, similar work on olive leaves subjected to UV-B stress [84] and a paper on Micro-Tom leaves subjected to heat stress [85] can be cited. In both cases, the stress treatment altered the profile of RuBisCO isoforms resulting in a more targeted use of isoforms, those most capable of functioning in the altered environmental conditions.

### 2.7. Pisanello Cultivar Exhibits the Most Consistent Increase in Sucrose Synthase

**Figure 7:**
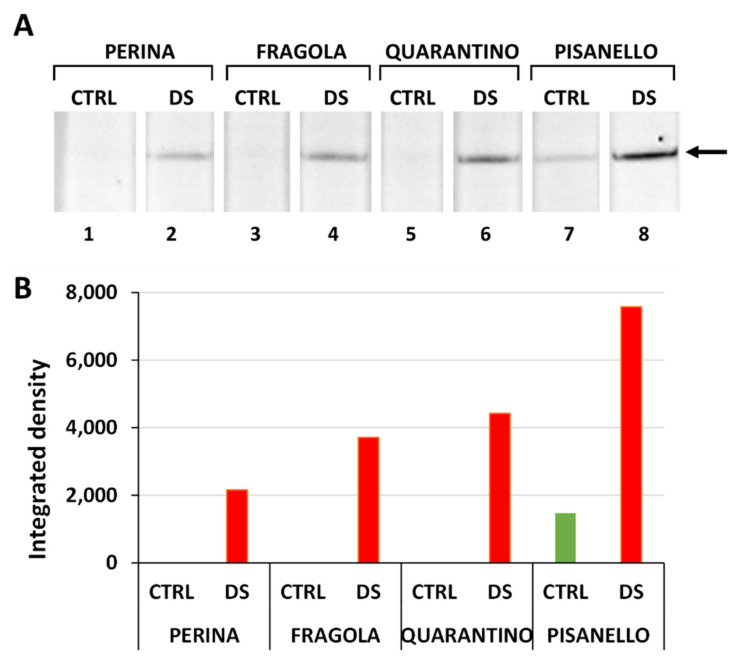
Content of sucrose synthase (SuSy) in the leaves of the four cultivars, both in control (CTRL) and in stressed samples (DS). (**A**) Immunoblotting; the arrow indicates the position of the cross-reactive SuSy. Lane 1, Perina CTRL; lane 2, Perina DS; lane 3 Fragola CTRL; lane 4, Fragola DS; lane 5, Quarantino CTRL; lane 6 Quarantino DS; lane 7, Pisanello CTRL; lane 8, Pisanello DS. (**B**) Quantitative analysis of immunoblotting to SuSy. It should be noted that the level of SuSy in the control samples of Perina, Fragola and Quarantino was extremely low, almost indistinguishable from the background.

SuSy is a key enzyme in sucrose metabolism as it cleaves sucrose producing UDP-glucose and fructose. While fructose can be directed toward respiration, UDP-glucose provides a conservative form of energy that can be redirected toward both intracellular metabolic processes and in the building of cell wall polysaccharides [86]. Thus, a consistent change in the amount or activity of SuSy impacts multiple aspects of cellular physiology. As can be seen in Figure 7A, the content of SuSy increases in plants subjected to water deprivation, compared to plants normally irrigated. In addition, the signal intensity of SuSy (Figure 7B) as detected in drought-stressed plants increased significantly in the most susceptible cultivar (Pisanello) than in those more tolerant. These results are consistent with findings in the literature; as SuSy catalyzes the cleavage of sucrose into its hexose monomers (UDP-glucose and fructose), more susceptible cultivars may have a more pressing need to positively regulate SuSy expression in order to increase the levels of free sugars, which act as osmoprotectants under osmotic stress conditions [48]. A direct correlation between water deficiency and sucrose synthase was also observed in selected species of the genus Populus. Although levels of soluble sugars did not show a direct correlation with increased sucrose synthase, it was evident that sucrose synthase increased in response to a water-deficient condition [87].

Indeed, the accumulation of Susy can also be related to an increased production of fructose that accumulates in plants under drought stress, like in wheat [88]. However, it is worth noting that drought stress does not always result in an increase in sucrose synthase content. For example, in wheat seedlings undergoing water shortage both invertase and sucrose phosphate synthase increase in response to drought, whereas sucrose synthase levels are unaffected between tolerant and susceptible plants [89]. An increase in sugar content and enzyme activity (such as sucrose phosphate synthase, sucrose synthase, and acid invertase) was also observed in soybean cultivars subjected to drought stress. Simultaneously, a decrease in starch, fructose, and glucose content and a parallel increase in sucrose content were found. This supports the evidence that an increase in sucrose-metabolizing enzymes does not necessarily result in a reduction in disaccharide levels [90]. In the roots (thus not in the leaves) tomato plants are reported to compensate for reduced energy production by targeting the sucrose synthase pathway, which is more energy conservative [91]. It must also be considered the hypothesis that increased sucrose cleavage by SuSy results in higher levels of UDP-glucose, the latter directed toward the synthesis of trehalose, a much-studied component in abiotic stress resistance [92,93].

SuSy is just one of the many enzymes regulated by phosphorylation events [94]. In general, phosphorylation/dephosphorylation mechanisms mediated by kinases and phosphatases control numerous metabolic enzymes as well as proteins involved in signal transduction. Indeed, metabolic adaptations are very delicate processes that must be finely regulated [95]. It follows that the activity of proteins examined in this work might depend on their regulation by phosphorylation in addition to their relative content. For this reason, we carried out a preliminary analysis by determining the changes in protein phosphorylation in the leaves of the four tomato cultivars (Figure S1). Phosphorylation levels were analyzed after separation of proteins on gel, transfer to membrane, and staining with a phosphoamino acid specific dye. We found differences in the phosphorylation levels of proteins expressed in drought-stressed plants compared to controls. The result is consistent with what Raghavendra and coworkers [95] found in tomato, where protein phosphorylation levels change under drought stress conditions. The Perina cultivar is characterized by slight changes in protein phosphorylation levels after drought stress; this would confirm that the Perina cultivar is the most tolerant to drought stress as it does not require major post-translational protein modifications to increase drought tolerance. In contrast, the Fragola and Quarantino cultivars show significant changes in protein phosphorylation levels in the water-deprived sample compared to control. Finally, in the Pisanello cultivar phosphorylation levels are drastically reduced in samples subjected to drought stress compared to control samples. This could suggest that effective responses against drought stress involve adequate protein phosphorylation mechanisms that the Pisanello cultivar is unable to implement.

### 2.8. Sucrose, Glucose, and Fructose Increase Differentially in Drought-Stressed Cultivars

Carbohydrates produced by photosynthesis in plant leaves provide energy and building blocks for growth and productivity. In addition to their energetic action, soluble carbohydrates (e.g., sucrose, fructose, glucose) act as important osmoregulatory substances capable of maintaining cell turgor under conditions of osmotic stress (such as that caused by drought and salt stress) [96]. Therefore, the regulation of soluble carbohydrate concentrations is an important adaptation of plants to water deficit. 

**Figure 8:**
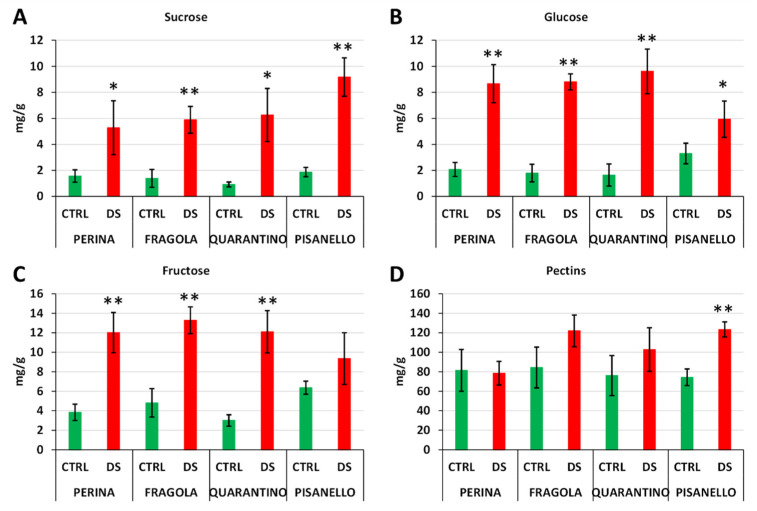
Content in mg per g of sucrose (**A**), glucose (**B**), fructose (**C**) and water-soluble pectins (**D**) in leaves sampled from control (green bars) and drought stressed (red bars) plants belonging to four Tuscan tomato cultivars. Asterisk indicates significant difference between control and stressed plants with *p* ≤ 0.05 (*) or *p* ≤ 0.01 (**).

Sucrose is the main product of reactions involving 3-carbon sugars generated by photosynthesis and represents a form of energy storage and transport [47]. Figure 8A shows the increase in sucrose in DS cultivars as well as the higher amount of sucrose in the Pisanello cultivar than in Perina or Fragola. In contrast, dissimilar data were obtained for glucose (Figure 8B) and fructose (Figure 8C), respectively. Again, we observed an increase in these two carbohydrates in drought-stressed plants but, unlike sucrose, Pisanello was the cultivar with the lowest amount of glucose and fructose. In contrast, the cultivars Perina, Fragola, and Quarantino showed a significant increase in both sugars.

In addition to being cleaved into glucose and fructose, sucrose can be cleaved into fructose and UDP-glucose by sucrose synthase (SuSy) [47]. In leaves, sucrose levels are also influenced by biosynthesis activity. Therefore, a direct correlation between drought stress tolerance and sucrose level is not straightforward. As described above, sucrose cleavage by SuSy has the advantage of conserving some of the energy of sucrose, which has significant implications in recovery from stress conditions [89]. It should also be considered that most stress conditions (especially drought) result in carbohydrate accumulation in leaves, which may play a key role in osmoprotection and osmotic adaptation [97]. Thus, in this case, it can be assumed that the more tolerant cultivars attempt to break down sucrose to have more available osmoprotectants. In contrast, the tolerance mechanism of the Pisanello cultivar is not as efficient because Pisanello continues to produce sucrose while also hypothetically reducing the synthesis of osmoprotectants, at the same time gaining less energy to counteract the effects of stress.

Because the content of sucrose and related sugars in leaves is the result of different metabolic pathways, the data on sugar content do not perfectly match the expression of SuSy (Figure 7). In particular, Pisanello, while showing an increase in SuSy, does not exhibit a comparable increase in free sugars, such as fructose and glucose, which would also be excellent osmoprotectants. Consequently, the increased content of SuSy does not always correspond to a direct cleavage of sucrose. We can speculate that the increase in SuSy does not imply higher enzyme activity. This could be related to the lower levels of phosphorylation observed in Pisanello.

The analysis of soluble sugars in tomato cultivars was also extended to water-soluble pectins (Figure 8D). Generally, drought stress conditions can impact cell wall composition. Although it is challenging to draw a general picture, water deficiency induces cell wall strengthening through increased production of hemicelluloses and reduced activity of pectin-degrading enzymes such as polygalacturonases [98]. The latter finding is not constant; indeed, in cucumber conditions of water stress induce an increase in the expression of the polygalacturonase gene and therefore probably a higher degradation of pectins [99]. Strengthening of the cell wall could allow the cells to counteract the loss of water and to maintain an adequate level of turgidity even at low water potential. This is coupled with increased pectin biosynthesis, sometimes even increased branching resulting in enhanced binding to water molecules, as well as improved cross-linking with other polysaccharides [100]. Nor can it be excluded that a remodeling of pectins can be perceived as a signal of stress conditions and initiate response mechanisms [101]. Figure 8D shows an increase in water-soluble pectins in the cultivars Fragola, Quarantino, and Pisanello, but not in the cultivar Perina. However, in both Fragola and Quarantino differences are not statistically significant. First, this suggests that each cultivar performs differently in terms of soluble pectin production. In addition, the finding implies that the most tolerant cultivars (Fragola, Perina, and Quarantino) do not need to increase the level of water-soluble pectins, a fact that could contribute to its tolerance to drought stress. However, a significant increase in water-soluble pectins is only found in the Pisanello cultivar, the most susceptible among those examined. This might suggest that the damage observed in the Pisanello cultivar is also due to excessive production and release of water-soluble pectins.

## 3. Materials and Methods

### 3.1. Growth Conditions of Tomato Plants and Stress Treatment

The plants studied in the present work are a subset of the nine tomato cultivars previously analyzed [54] from a morpho-physiological point of view; therefore, plants followed their same growth and drought stress conditions hereby briefly summarized. For each cultivar, 10 plants were studied during the vegetative phase, five plants were used as control (CTRL) and five subjected to drought stress (DS). The stress condition was maintained for 16 days and consisted in complete watering withdrawal; the CTRL group was kept in a fully irrigated regime for the whole period. From the morpho-physiological results it was possible to identify the following four cultivars of interest.

- Perina and Fragola, the most tolerant cultivars,
- Quarantino, the cultivar with medium tolerance,
- Pisanello, the most susceptible.

The analysis in this manuscript was carried out on the above four cultivars. Biochemical aspects related to proteins involved in the defense mechanisms against drought stress were investigated. All samples were taken at the final stress phase (after 16 days of drought stress). The timing of drought stress was chosen according to [49,53,102,103]. Samples were immediately placed at −80 °C (ThermoFisher, TSZ400VGP, Suzhou, Jiangsu, China) and stored for 3 months for subsequent analysis.

### 3.2. Protein Extraction

Protein extraction was performed as described by Faurobert and coworkers [104]. Leaves were ground in liquid nitrogen, 1 g of sample was weighed and resuspended in 3 mL of Extraction Buffer (500 mM Tris-HCl, 50 mM EDTA, 700 mM sucrose, 100 mM KCl, 2% ß-mercaptoethanol and 1 mM of protease inhibitors, pH 8.0). Samples were vortexed and incubated on ice for 10 min with gentle agitation to allow for sample resuspension. An equal volume of Tris-buffered phenol (Amresco-Interchim, Biotechnology Grade) was then added, vortexed for 3–5 min and incubated for 10 min at room temperature (RT) with gentle agitation. The mixture was centrifuged at 5500× *g* for 10 min at 4 °C and the upper phase was taken to which 3 mL of Extraction Buffer were added; samples were vortexed for 3 min and centrifuged at 5500× *g* for 10 min at 4 °C. The upper phase was collected and supplemented with four volumes of precipitation solution (0.1 M ammonium acetate in methanol), mixed by inversion, and incubated at −20 °C for at least 4 h or overnight. The mixture was centrifuged at 5500× *g* for 10 min at 4 °C and the supernatant was removed. The pellet was washed with the precipitation solution, centrifuged at 5500× *g* for 5 min at 4 °C and the supernatant was removed. This last step was repeated twice. The last pellet was washed with cold acetone, centrifuged at 5500× *g* for 5 min at 4 °C and the supernatant was removed. Samples were then dried at RT under a fume hood for 10 min. Afterwards, 100 μL of 0.2 M NaOH were added and samples incubated for 2 min for more effective solubilization. A volume of 200 μL of LSB1X for 1-D electrophoresis and 200 μL of Rehydration Buffer (RB) for 2-D electrophoresis were added to the samples. Finally, samples were centrifuged for 15 min at 10,000× *g* at RT, the supernatants were collected, and the protein concentration was calculated using the 2-D Quant Kit (GE, USA).

### 3.3. 1-D Electrophoresis and Immunoblotting

Electrophoresis was conducted on 10% bis-Tris SDS-PAGE [105] at pH 6.5–6.8. Volumes containing 30 μg of protein from the CTRL and DS samples of the four cultivars were loaded into each gel. Electrophoresis was carried out on a Criterion cell (Bio-Rad Laboratories, Segrate, Italy) equipped with a Power Pac BioRad 300 at 200 V for approximately 45 min. XT MOPS (Bio-Rad Laboratories, Hercules, CA, USA) was used as a running buffer. Transfer of proteins from gels to nitrocellulose or PVDF (for osmotin and dehydrins) membranes was performed using a Trans-Blot Turbo Transfer System (Bio-Rad Laboratories, Segrate, Italy) according to the manufacturer’s instructions (using the setting for low molecular weight proteins). After blotting, membranes were blocked overnight at 4 °C in 5% ECL Blocking Agent (GE HealthCare Dornstadt, Germany) with 0.1% Tween-20 in TBS (20 mM Tris pH 7.5, 150 mM NaCl). After washing with 1X TBS, membranes were incubated with the primary antibody for 1 h. Below is a list of the antibody used:

- The Anti-HSP70 (ADI-SPA-820-D) was a mouse monoclonal type antibody and was diluted 1:5000 (Enzo Life Sciences). This antibody was purified from human HeLa cells. It recognizes protein homologues in plants and its efficiency has been confirmed in *Citrus* L. and pepper plants [106] as well as in leaves of olive trees [84].
- The Anti-Cyclophilin (Anti-CYP) was a rabbit polyclonal antibody and was diluted 1:3000 [107]. This antibody was raised against a 172-residue polypeptide of *Solanum sogarandinum* O. [108]. Our workgroup has also successfully tested it on *Pyrus* L. pollen [107].
- The Anti-Dehydrin (AS07 206A) was a rabbit polyclonal antibody and was diluted 1:1000 (Agrisera). This antibody binds to the dehydrin family, which are proteins involved in protective reactions against dehydration. Specifically, the antibody binds to the k-segment peptide sequence (TGEKKGIMDKIKEKLPGQH) conserved in a wide range of different plant species. The reactivity of this antibody has also been confirmed in *Solanum licopersicom* L., as well as in other species such as *Pistacia vera* L. and *Cucumis sativus* L. [109,110,111].
- The Anti-Osmotin (AS19 4336) was a rabbit polyclonal antibody and was diluted 1:1000 (Agrisera). This antibody was derived from the *Nicotiana tabacum* L. protein sequence, ranging from amino acid 22 to 246. The predicted reactivity is also on *Solanum lycopersicum* L.
- The Anti-Aquaporins (AS09 489) was a rabbit polyclonal antibody and was diluted 1:1000 (Agrisera). The immunogen for aquaporin antibody is a KLH-conjugated synthetic peptide derived from N terminus of *Raphanus sativus* L. The peptide is conserved in PIP1;1, PIP1;2, PIP1;3 N-terminus of *Raphanus sativus* L. and in all 5 isoforms (PIP1;1, PIP1;2, PIP1;3, PIP1;4, PIP1;5) of *Arabidopsis thaliana* L. The reactivity in *Solanum lycopersicum* L. is not confirmed but predicted.
- The Anti-RuBisCO was a rabbit polyclonal antibody and was diluted 1:10,000 (Agrisera). The immunogen for the RuBisCO antibody was a synthetic KLH-conjugated peptide preserved in all known plant, algal and cyanobacterial protein sequences. Reactivity was confirmed and predicted on several plant species but not on *Solanum lycopersicum* L. However, the reactivity against tomato was evaluated in a previous work on the Micro-Tom cultivar [85].
- The K4 anti-SuSy was a rabbit polyclonal antibody and was diluted 1:1000 [112]. The antibody against SuSy was made in the *Zea mays* L. on the complete protein [113].

Subsequently, membranes were washed twice with 1X TBS and then incubated with peroxidase-conjugated secondary antibodies for 1 h. The Anti-rabbit IgG (Bio-Rad, #1706515) and Anti-mouse IgG (Bio-Rad, #1706516) were polyclonal antibodies and were diluted 1:3000. After rinsing the membranes with 1X TBS, the immunological reactions were visualized with ClarityTM Western ECL Substrate (Bio-Rad Laboratories, United State). Images of blots were acquired using a Fluor-S apparatus (Bio-Rad Laboratories, Segrate, Italy) and analyzed with the Quantity One software (Bio-Rad Laboratories, Segrate, Italy). Finally, densitometric analysis was performed with the same software for a relative quantitative evaluation of band intensity (expressed as Integrated density).

### 3.4. 2-D Electrophoresis and Immunoblotting of RuBisCO

2D electrophoresis and immunoblotting on RuBisCO were performed as described in [84,85]. Briefly, samples were supplemented with 18 mM DTT and 10% IPG Buffer, then brought to the volume of 200 µL with RB to obtain a protein concentration of 1.5 mg/mL. Samples were loaded into the Immobiline DryStrip Reswelling Tray (Pharmacia Biotech) and Readystrip IPG pH 5-8 (Bio-Rad) were placed on top of samples. After 30 min, strips were covered with mineral oil (Bio-Rad) and allowed rehydrating for 24 h. Strips were then positioned on the Focusing Tray (Bio-Rad) and were covered with mineral oil; the tray was positioned in the Protean IEF Cell (Bio-Rad) and run was carried out at 20 °C following an increasing voltage program: from 0 to 500 V in 1 h, 500 V constant for 1 h, from 500 V to 4000 V in 2 h, 4000 V for 2 h, from 4000 V to 8000 V in 2 h, 8000 V constant up to 15,000 V/hour, from 8000 V up to 500 V in 30 min and 500 V until strips were removed. For separation in the second dimension, strips were washed with Equilibration Buffer 1 (130 mM DTT, 6 M Urea, 2% SDS, 0.375 M Tris-HCl pH 8.8 and 20% glycerol) for 10 min and then with Equilibration Buffer 2 (130 mM Iodoacetamide, 6 M Urea, 2% SDS, 0.375 Tris-HCl pH 8.8 and 20% glycerol) for 10 min. At the end, strips were placed in the well of 10% Criterion XT PreCast gel (Bio-Rad) and immobilized with agarose gel. The electrophoretic run was performed in a Criterion Cell (Bio-Rad) at 200 V for 1 h using XT MOPS buffer (Bio-Rad). Gels were transferred to nitrocellulose membranes for immunoblotting. The membranes were blocked overnight at 4 °C with 5% ECL Blocking Agent (Bio-Rad) in TBS (20 mM Tris pH 7.5, 150 mM NaCl) plus 0.1% Tween-20. Membranes were incubated for 1 h at RT with a primary anti-RuBisCO antibody, diluted 1:10,000 (Agrisera). After washing in 1X TBS, membranes were incubated for 1 h with a secondary goat anti-rabbit antibody, diluted 1:3000 and conjugated to peroxidase. Visualization of the immunological reaction was performed using ClarityTM Western ECL Substrate (Bio-Rad Laboratories, United State). Images of blots were acquired using a Fluor-S apparatus (Bio-Rad Laboratories, Segrate, Italy) controlled by Quantity One software (Bio-Rad). For the comparison of immunoblots, the PDQuest software (Bio-Rad, version 8.0) was used.

### 3.5. Analysis of Soluble Sugars

High Pressure Liquid Chromatography (HPLC) was used for the analysis of sugars (pectins, sucrose, fructose, and glucose) [85,114]. Briefly, 100 mg of leaf samples powdered with liquid nitrogen were added to 1 mL of distilled H_2_O. Samples were homogenized by Ultra-Turrax^®^ T-25 basic (IKA^®^-Werke GmbH & Co. KG, Staufen im Breisgau, Germany), centrifuged at 3000 RCF for 5 min; the supernatants were transferred to 2 mL Eppendorf^®^ tubes and then centrifuged again at 12,000 RCF for 5 min (Eppendorf^®^ Microcentrifuge 5415D, Hamburg, Germany). Samples were filtered (0.45 µm) and 20 µL of each extract was injected into a Waters Sugar-Pak I ion exchange column (6.5 × 300 mm) at a temperature of 90 °C. The mobile phase consisted of MilliQ H_2_O (pH 7) with a flow of 0.3 mL min^−1^. The overall duration of the separation was 30 min. Identification of components was done using a Waters 2410 refractive index detector by comparing the retention times with those of reference standards. The experiment was conducted in three technical replicates for each sample. Finally, the mean and standard deviation were calculated. To verify the significance of the data obtained, the *t*-test (* *p* ≤ 0.05, ** *p* ≤ 0.01) were carried out.

### 3.6. Phosphoprotein Profiling

Pro-Q^®^ Diamond Blot Reagent & Buffer (Thermo Fisher) was used to highlight the phosphoprotein patterns according to the protocol provided by the company. Proteins were separated by electrophoresis and then transferred to PVDF membranes (pre-moistened in methanol). After electroblot, membranes were allowed to dry completely. Proteins were fixed on membranes by dipping it face down in 25 mL of Fix Solution (7% acetic acid, 10% methanol) for 10 min. Membranes were washed by immersion in 25 mL of dH_2_O for 5 min (three times). Proteins were stained by immersing the membrane in 25 mL of the diluted Pro-Q^®^ Diamond Phosphoprotein Blot solution for 15 min. Membranes were destained by washing in 30 mL of Destain solution (50 mM sodium acetate, pH 4.0, 20% acetonitrile) for 5 min (three times). Fluorescent phosphoproteins could be visualized by the Fluor-S apparatus (Bio-Rad Laboratories, Segrate, Italy) by illuminating membranes with UV light using a 615 nm bandpass filter; exposure times were 10–30 s. The resulting electrophoretic lanes were scanned by Quantity One software (Bio-Rad).

## 4. Conclusions

In this work, four Tuscan tomato cultivars were characterized using a panel of biochemical analyses. These revealed critical differences among cultivars in the response to drought. Some mechanisms, such as increased levels of HSP70 and cyclophilins, are common and implemented by all cultivars, albeit with some differences among cultivars (e.g., Perina increases HSP70 content more than Pisanello or does not make use the same cyclophilins). These data confirm the important protective role of HSP70 and cyclophilins in the correct folding of proteins. In fact, previous works on the morpho-physiological aspects of the above cultivars already indicated Perina as the most tolerant, while Pisanello was the most susceptible.

Dehydrin and osmotin contents are higher when plants are severely affected by drought stress. While dehydrins are substantially expressed by all cultivars under stress, osmotins are found only in Pisanello. This can be explained by the fact that the susceptible cultivar Pisanello shows more damage to the photosynthetic system. Therefore, it is the only cultivar that requires expression of osmotin, which plays a role in chlorophyll protection. Analysis on RuBisCO corroborated this hypothesis. In fact, a drastic decrease in RuBisCO content is shown in the cultivar Pisanello under drought stress. Moreover, the 9905 isoform of RuBisCO is apparently typical of the most tolerant cultivars (Perina and Fragola) but not of the Pisanello cultivar. RuBisCO is an enzyme with several co/post-translational modification sites; therefore, under stress these modifications may generate isoforms that are more suitable to counteract a demanding situation such as drought. This concept is further supported by the evidence that Pisanello, compared to other cultivars, shows a pronounced generic dephosphorylation pattern. Phosphorylation/dephosphorylation may contribute to an increase or decrease in sucrose synthase (SuSy) activity. The content of this enzyme increases in all stressed cultivars, especially in Pisanello. SuSy allows the cleavage of sucrose into fructose and UDP-glucose, thus feeding the biosynthesis pathway of osmoprotective sugars. However, the Pisanello cultivar still produces sucrose in large quantities without breaking it down into glucose and fructose, which could instead be more useful during stress.

In conclusion, this study confirmed at the biochemical level the results previously obtained by morpho-physiological analyses on the tolerance or susceptibility of tomato cultivars to drought stress. More specifically, we have examined the biochemical mechanisms that are activated by drought and that increase stress tolerance. From this and previous studies, the Perina cultivar is confirmed as the most tolerant, because it can activate all the mechanisms necessary for tolerance. Most importantly, it can keep the photosynthetic system active by selecting the best RuBisCO isoforms and increasing the content of aquaporins, beneficial for the transport of CO_2_ and H_2_O.

## Data Availability

Not applicable.

## Supplementary Materials

The following supporting information can be downloaded at: https://www.mdpi.com/article/10.3390/ijms23105412/s1.
