# Supplementary figures and images for: Distinct Tomato Cultivars Are Characterized by a Differential Pattern of Biochemical Responses to Drought Stress
Source: Int J Mol Sci. 2022 May 12;23(10):5412. doi: 10.3390/ijms23105412 (PMC9141555; doi:10.3390/ijms23105412)

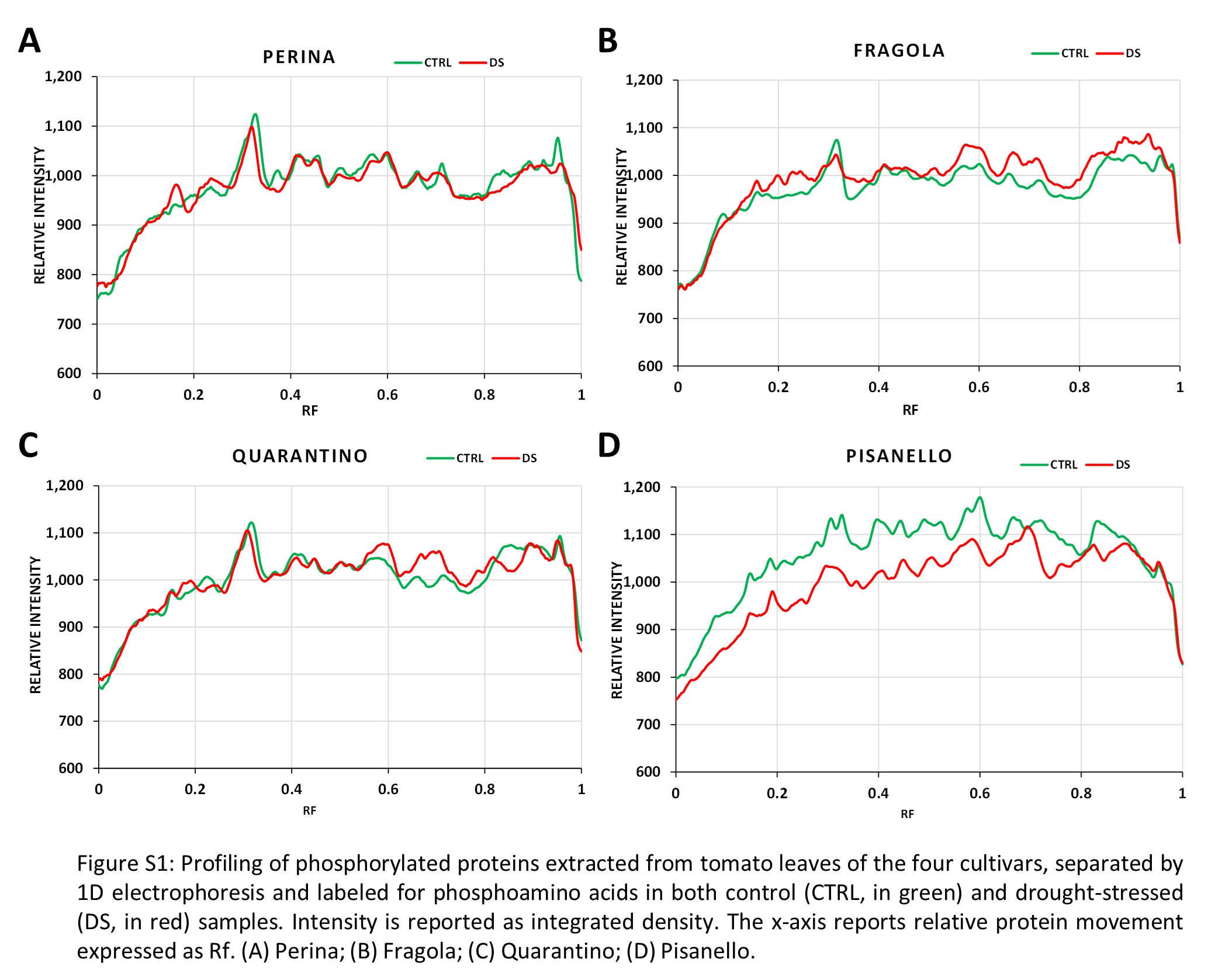

Supplement: Supplementary file 1 [file ijms-23-05412-s001.zip › ijms-1691422-supplementary.tif]
